# *In Utero* and Lactational Exposure to PCBs in Mice: Adult Offspring Show Altered Learning and Memory Depending on *Cyp1a2* and *Ahr* Genotypes

**DOI:** 10.1289/ehp.1002965

**Published:** 2011-05-13

**Authors:** Christine P. Curran, Daniel W. Nebert, Mary Beth Genter, Krishna V. Patel, Tori L. Schaefer, Matthew R. Skelton, Michael T. Williams, Charles V. Vorhees

**Affiliations:** 1 Department of Environmental Health and Center for Environmental Genetics, University of Cincinnati Medical Center, Cincinnati, Ohio, USA; 2 Department of Pediatrics, Division of Neurology, University of Cincinnati Medical Center and Cincinnati Children’s Research Foundation, Cincinnati, Ohio, USA

**Keywords:** acoustic startle response, aryl hydrocarbon receptor (AHR), coplanar PCBs, cytochrome P450 1A2 (CYP1A2), developmental neurotoxicity, locomotor activity, long-term potentiation, Morris water maze, noncoplanar PCBs, novel object recognition, PCB exposure *in utero*, PCB exposure via breast milk, polychlorinated biphenyls (PCBs), prepulse inhibition

## Abstract

Background: Both coplanar and noncoplanar polychlorinated biphenyls (PCBs) exhibit neurotoxic effects in animal studies, but individual congeners do not always produce the same effects as PCB mixtures. Humans genetically have > 60-fold differences in hepatic cytochrome P450 1A2 (CYP1A2)-uninduced basal levels and > 12-fold variability in aryl hydrocarbon receptor (AHR)affinity; because CYP1A2 is known to sequester coplanar PCBs and because AHR ligands include coplanar PCBs, both genotypes can affect PCB response.

Objectives: We aimed to develop a mouse paradigm with extremes in *Cyp1a2* and *Ahr* genotypes to explore genetic susceptibility to PCB-induced developmental neurotoxicity using an environmentally relevant mixture of PCBs.

Methods: We developed a mixture of eight PCBs to simulate human exposures based on their reported concentrations in human tissue, breast milk, and food supply. We previously characterized specific differences in PCB congener pharmacokinetics and toxicity, comparing high-affinity–AHR *Cyp1a2* wild-type [*Ahr^b1^_Cyp1a2*(+/+)], poor-affinity–AHR *Cyp1a2* wild-type [*Ahr^d^_Cyp1a2*(+/+)], and high-affinity–AHR *Cyp1a2* knockout [*Ahr^b1^_Cyp1a2*(–/–)] mouse lines [Curran CP, Vorhees CV, Williams MT, Genter MB, Miller ML, Nebert DW. 2011. In utero and lactational exposure to a complex mixture of polychlorinated biphenyls: toxicity in pups dependent on the *Cyp1a2* and *Ahr* genotypes. Toxicol Sci 119:189–208]. Dams received a mixture of three coplanar and five noncoplanar PCBs on gestational day 10.5 and postnatal day (PND) 5. In the present study we conducted behavioral phenotyping of exposed offspring at PND60, examining multiple measures of learning, memory, and other behaviors.

Results: We observed the most significant deficits in response to PCB treatment in *Ahr^b1^_Cyp1a2*(–/–) mice, including impaired novel object recognition and increased failure rate in the Morris water maze. However, all PCB-treated genotypes showed significant differences on at least one measure of learning or behavior.

Conclusions: High levels of maternal hepatic CYP1A2 offer the most important protection against deficits in learning and memory in offspring exposed to a mixture of coplanar and noncoplanar PCBs. High-affinity AHR is the next most important factor in protection of offspring.

Polychlorinated biphenyls (PCBs) are among the top five priority pollutants (Agency for Toxic Substances and Disease Registry 2007). The primary route of human exposure is consumption of contaminated foods (Huwe and Larsen 2005); in the past, occupational exposures were significant (Gustavsson and Hogstedt 1997). Populations near polluted toxic waste dump sites have demonstrated learning, memory, and behavioral abnormalities in children exposed *in utero* and via breast milk (Schantz et al. 2003). Therefore, previous studies have defined at-risk populations, primarily based on their exposure to PCB-contaminated foods or their proximity to PCB-contaminated sites.

Evidence for PCB-induced neurotoxicity includes studies of exposed human populations worldwide (Grandjean et al. 2001; Guo et al. 1997; Gustavsson and Hogstedt 1997; Jacobson and Jacobson 1997, 2003; Nakai et al. 2004). These studies consistently show learning, memory, and behavioral deficits that extend into school age (Jacobson and Jacobson 2003; Vreugdenhil et al. 2004) and increased neurodegenerative diseases (Petersen et al. 2008; Schantz et al. 2001). The greatest risk is to children exposed *in utero* and through consumption of contaminated breast milk (Guo et al. 2004; Schantz et al. 2003). Studies in nonhuman primates (Rice 2000; Schantz et al. 1989) and rodents (Gilbert et al. 2000; Roegge and Schantz 2006) have confirmed the unique susceptibility of the developing central nervous system (CNS) to PCBs.

In the present study we used a previously developed mixture of eight PCBs that included coplanar and noncoplanar PCBs prevalent in food, human tissue, and breast milk (Curran et al. 2011); these PCBs were chosen because they have previously been implicated in developmental neurotoxicity. Single-congener studies offer utility when searching for mechanisms, but they are less satisfactory at modeling human exposures.

Coplanar PCBs are aryl hydrocarbon receptor (AHR) ligands (Poland and Glover 1977), and maternal levels of hepatic cytochrome P450 1A2 (CYP1A2) influence the amount of AHR ligand reaching the embryo or fetus (see Dragin et al. 2006 and references therein). Moreover, humans are known genetically to exhibit > 12-fold variability in AHR affinity and > 60-fold differences in hepatic CYP1A2 basal uninduced levels (Nebert et al. 2004). Thus, we administered the PCB mixture to mice representing extremes for variation in high- versus poor-affinity AHR and high versus absent CYP1A2 basal levels.

In characterizing these mice (Curran et al. 2011), we examined effects of the PCB mixture [given on gestational day (GD) 10.5 and postnatal day (PND) 5] on three genotypes: wild-type having high-affinity AHR [*Ahr^b1^_Cyp1a2*(+/+)], wild-type having poor-affinity AHR [*Ahr^d^_Cyp1a2*(+/+)], and knockout having high-affinity AHR [*Ahr^b1^_Cyp1a2*(–/–)]. These lines were evaluated for PCB effects on birth weight, growth, immunosuppression, AHR activation, and CYP1A1 and CYP1A2 mRNA levels in tissues of the mother, embryo, fetus, and pup; the concentrations of each of the PCB congeners in these tissues were measured at five time points. We also confirmed important genetic differences in the above-mentioned parameters (Curran et al. 2011). In that study (Curran et al. 2011), administration of the mixture to the mother at GD10.5 and PND5 resulted in continuous AHR activation in the high-affinity–*Ahr^b1^* embryo, fetus, and weanling. GD10.5 to PND20 is the period of rodent brain development that most closely matches brain development in the second to third trimesters of human development (Clancy et al. 2007).

## Materials and Methods

*Chemicals.* Noncoplanar PCB congeners 105, 118, 138, 153, and 180 and coplanar  PCB congeners 77, 126, and 169 [see Supplemental Material, Table S1 (http://dx.doi.org/10.1289/ehp.1002965)] were purchased from ULTRA Scientific (North Kingstown, RI) and dissolved in acetone and corn oil (acetone removed under argon). Other reagents were from Fisher (Fairlawn, NJ) or Sigma (St. Louis, MO). Personnel were instructed in safe handling and disposal of PCBs.

*Animals.* Mice (Table 1) included C57BL/6J (B6) and B6.D2-*Ahr^d^* (congenic having poor-affinity *Ahr^d^* allele from DBA/2J) from Jackson Laboratory (Bar Harbor, ME); both are *Cyp1a2*(+/+) wild-type. The *Ahr^b1^_Cyp1a2*(–/–) knockout mouse is an in-house line (Liang et al. 1996). Backcrossing produced genotypes that express > 99.8% B6. Animals were housed in a vivarium accredited by the Association for Assessment and Accreditation of Laboratory Animal Care; the animals were treated humanely and with regard for alleviation of suffering.

*Breeding.* Nulliparous females 3–5 months of age (body weight, 20–25 g) were used for all matings. The morning when a vaginal plug was found was considered GD0.5, and plug-positive females were removed from the breeding cages. Pregnant females were housed individually with pups until weaning on PND28.

*Dosing of animals.* Pregnant females were given the PCB mixture by gavage on GD10.5 and PND5; these time points were chosen to ensure continual AHR activation throughout lactation and were based on our previous study (Curran et al. 2011). Controls were gavaged with an equivalent volume of corn oil vehicle (15 mL/kg). Dosing was delayed until GD10.5 to avoid interfering with implantation and to minimize neonatal lethality (Curran et al. 2006).

*Behavior.* Animals were tested in groups from all three genotypes (PCB-treated vs. corn-oil–treated controls). One male and one female per litter were tested (16–20/group) beginning on PND60: week 1, elevated zero maze, locomotor activity, and acoustic startle response (ASR) with prepulse inhibition (PPI); week 2, novel-object recognition; week 3, Morris water maze (MWM) cued; week 4, MWM hidden acquisition; week 5, MWM hidden reversal; week 6, MWM hidden shift; week 7, locomotor activity with (+) methamphetamine (1 mg/kg) challenge. Mice were placed in the apparatus for 30 min to habituate them to the environment; they were then removed, injected with methamphetamine, and returned to the apparatus for an additional 120 min. All tests were performed during the light portion of the light:dark cycle.

Elevated zero maze. The apparatus for this test is a circular runway (105-cm diameter), 72-cm above the floor with a 10-cm path divided into equal quadrants; two opposite quadrants have 28-cm walls, and two remaining opposite quadrants have 1.3-cm acrylic curbs. Mice were videotaped for 5 min. Time in open and numbers of head dips and zone crossings were scored (Shepherd et al. 1994).

Locomotor activity. For evaluation of locomotor activity, mice were tested for 1 hr in arenas that measured 41 × 41 cm and had 16 LED photocells in the *x*- and *y*-planes (Accuscan Instruments, Columbus, OH).

ASR-PPI. For this test, we used an SR-LAB apparatus (San Diego Instruments, San Diego, CA) with 5-min acclimation, followed by a 4 × 4 Latin square of four trial types repeated three times: no stimulus, startle signal (SS), 74-dB prepulse + SS, or 76-dB prepulse + SS. The intertrial interval was 8 sec, and the interstimulus interval was 70 msec. The signal was a mixed-frequency white noise burst (120 dB sound pressure level for 20 msec). Peak response amplitudes (*V*_max_) were analyzed.

Novel object recognition. For evaluation of novel object recognition, mice were habituated to arenas (91-cm diameter) for 2 days, followed by 2 days of exposure to two objects (10 min/day). On the test day new objects were presented until 30 sec of observation accrued; 1 hr later, the familiar (copy) and novel object were both presented, until 30 sec of observation accrued (up to 10 min).

MWM. The tank for the MWM was 122 cm in diameter (Vorhees and Williams 2006). Testing was as follows: day 1 consisted of six cued trials with the start and platform fixed; for days 2–6, there were two trials per day with random start and finish positions (curtains were closed to block visual cues). The 10-cm platform contained an orange ball 10 cm above the surface. Mice received three phases of hidden-platform testing, four trials per day for 6 days, with 30-sec probe trial on day 7 [see Supplemental Material, Table S2 (http://dx.doi.org/10.1289/ehp.1002965)]. Each phase used a smaller platform (10, 7, or 5 cm). Data were analyzed for latency, cumulative distance, path length, speed on platform trials and crossovers, average distance, and quadrant preference on probe. For additional information, see Supplemental Material, p. 2.

**Table 1:** Mouse lines and hypotheses regarding susceptibility or resistance to PCB-induced developmental neurotoxicity.

Genotype	AHR ligand	CYP1A2	Common name	Hypothesis
*Ahrd_Cyp1a2*(+/+)		Poor affinity		Present		B6.D2-*Ahrd*		Most resistant
*Ahrb1_Cyp1a2*(+/+)		High affinity		Present		C57BL/6J (B6)		Intermediate
*Ahrb1_Cyp1a2*(–/–)		High affinity		Absent		*Cyp1a2* knockout		Most susceptible

*Long-term potentiation (LTP).* We measured LTP using a MED64 multielectrode array (Alpha Med Sciences, Kadoma, Japan) (Shimono et al. 2002) on parasagittal hippocampal sections (350 μm) of PND30–35 mice. Paired pulses were delivered to CA1, and excitatory postsynaptic potentials (EPSPs) were recorded until stable. Slope of EPSPs was recorded for 90 min after a theta burst [tetanus = 100 Hz in 10 bursts (4 pulses/burst) delivered at a burst frequency of 5 Hz for 2 sec]. Sections were analyzed in duplicate for each animal.

*Monoamine neurotransmitter assay.* Neurotransmitters [dopamine (DA) and its metabolite 3,4-dihydroxyphenylacetic acid, and serotonin and its metabolite 5-hydroxyindoleacetic acid] were analyzed as previously described (Graham et al. 2011). See Supplemental Material, p. 4 (http://dx.doi.org/10.1289/ehp.1002965) for details.

*Corticosterone assay.* Corticosterone levels were measured using an immunoassay kit (Octeia Corticosterone EIA kit AC-14F1; IDS Inc., Fountain Hills, AZ) following the manufacturer’s protocol. Blood was collected in heparinized tubes and centrifuged at 2,500 relative centrifugal force for 5 min at 4°C, and plasma was stored at –80°C. All samples were run in duplicate, and corticosterone levels were calculated by comparison with a standard curve ranging from 0 to 133 ng/mL.

*Statistical analyses.* Behavioral data were analyzed using mixed-linear analysis of variance (ANOVA) with repeated measures or by analysis of covariance (ANCOVA) using SAS software (version 9.2; SAS Institute Inc., Cary, NC). Results were considered statistically significant if *p* < 0.05, as analyzed by slice-effect ANOVAs and biochemical data by two-way ANOVA followed by Holm-Sidak post hoc comparisons.

## Results

*Body weight.* Mice were weighed on PND60 and PND100; we observed no differences in body weight among treatment groups or genotypes.

**Figure 1:**
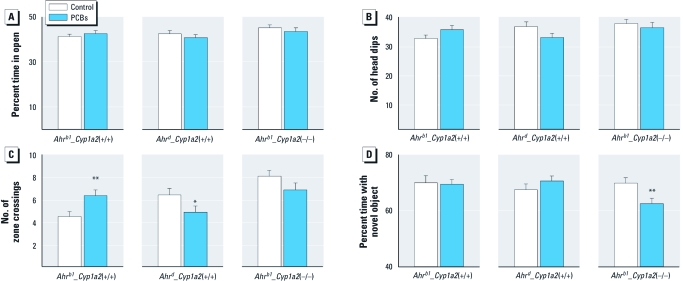
Results of elevated zero maze (5 min). (*A*) Percent time in open. (*B*) Number of head dips. (*C*) Number of zone crossings. (*D*) Percent time exploring novel object. Data shown are least-squares mean ± SE. *p < 0.05, and **p < 0.01, compared with untreated controls of the same genotype.

*Elevated zero maze.* PCB-treated *Ahr^d^_Cyp1a2*(+/+) mice exhibited fewer head dips and zone crossings (*p* = 0.06), indicating increased anxiety (Figure 1), whereas PCB-treated *Ahr^b1^_Cyp1a2*(+/+) mice showed more head dips (*p* = 0.09) and significantly more zone crossings than controls, suggesting a mild anxiety effect of PCBs but no differences in time in open (the principal index of anxiety in this test, which is fear of open spaces). Increased time in open therefore indicates decreased anxiety. The AHR phenotype caused opposite effects in response to PCBs, slightly increasing anxiety in poor-affinity–AHR mice (fewer head dips) and slightly decreasing anxiety in high-affinity–AHR mice. PCB decreased zone crossings in poor-affinity–AHR mice and increased them in high-affinity–AHR mice, suggesting that PCB causes increased anxiety in high-affinity–AHR mice. We cannot exclude that these differences contribute to variations in head dips; however, absence of difference in time in open (Figure 1A) argues against this interpretation.

*Novel object recognition.* PCB-treated *Ahr^b1^_Cyp1a2*(–/–) was the only group showing significant deficits in novel object recognition (Figure 1D). PCB-treated *Ahr^b1^_Cyp1a2*(–/–) mice spent a lower percentage of time exploring the novel object compared with controls, suggesting that PCB-treated *Ahr^b1^_Cyp1a2*(–/–) mice are less able to remember the familiar object and distinguish it from the new object.

**Figure 2:**
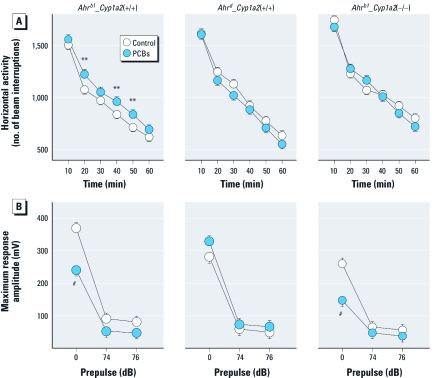
Results of locomotor activity (60 min; *A*) and ASR-PPI (*B*) tests (mean ± SE). **p < 0.01, and ^#^*p* < 0.001 compared with controls of the same genotype.

*Locomotor activity.* Decreases in PCB-treated rodent locomotor habituation have been reported (Eriksson 1997). Regardless of treatment, *Ahr^b1^_Cyp1a2*(–/–) mice were more active than the other genotypes (Figure 2A). Treatment differences were significant only for PCB-treated *Ahr^b1^_Cyp1a2*(+/+) mice, compared with controls during the middle (20-, 40-, and 50-min) intervals.

*ASR with PPI.* The ASR-PPI test measures baseline startle response and its attenuation when preceded by a lower-decibel tone preceding it (prepulse). We found no differences in PPI in PCB-treated mice compared with controls, regardless of AHR genotype. Untreated *Ahr^b1^_Cyp1a2*(–/–) mice had significantly reduced amplitude in ASR than did untreated controls of either of the other two genotypes. PCB-treated *Ahr^b1^_Cyp1a2*(–/–) mice exhibited decreased ASR (Figure 2B) compared with controls, as did the PCB-treated *Ahr^b1^_Cyp1a2*(+/+) mice. In contrast, PCB-treated *Ahr^d^_Cyp1a2*(+/+) mice showed a trend (*p* = 0.08) toward increased ASR. PCB exposure has been linked to hearing loss; however, hearing deficits cannot explain these results because all mice exhibited ASR after the 74- and 76-dB prepulses.

**Figure 3:**
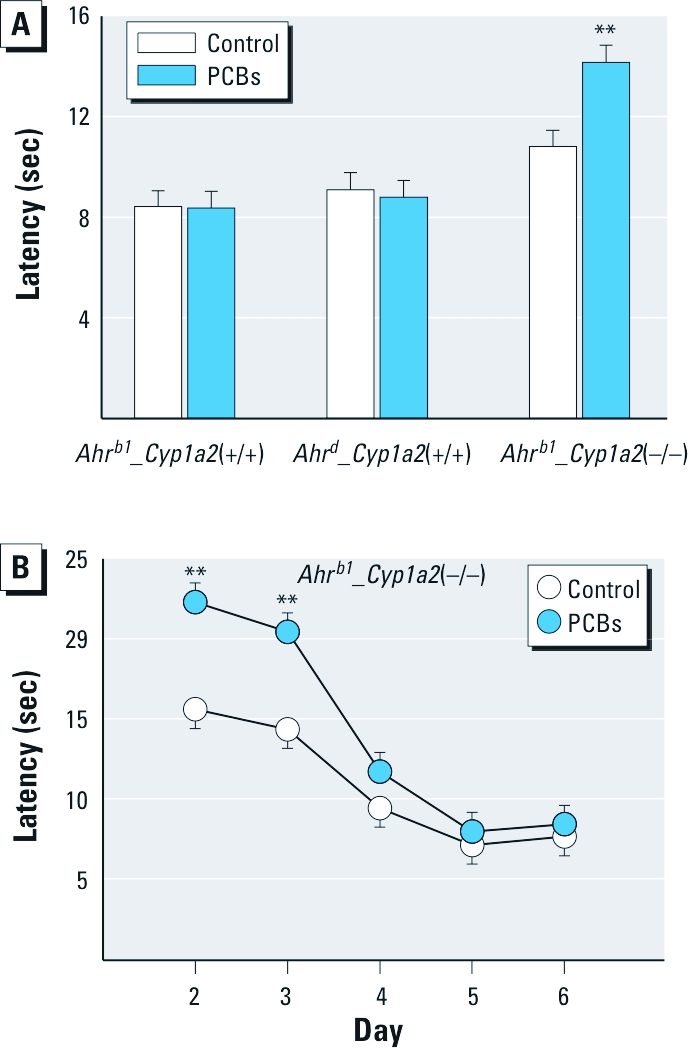
Results of the MWM (cued) test (mean ± SE). (*A*) Latency to reach goal. (*B*) Latency in the *Ahr^b1^_Cyp1a2*(–/–) group on days 2–6 (two trials per day). No differences were seen on day 1 (data not shown). **p < 0.01 compared with untreated control.

*MWM, cued platform.* We observed no differences among the groups on day 1 (data not shown). Data for days 2 and 3 showed that PCB-treated *Ahr^b1^_Cyp1a2*(–/–) mice took longer to reach the platform (greater latency) than did controls (Figure 3A). Further analysis revealed that this difference was attributable to slower swimming in PCB-treated *Ahr^b1^_Cyp1a2*(–/–) mice on days 2 and 3 (Figure 3B), with no significant differences thereafter.

**Figure 4:**
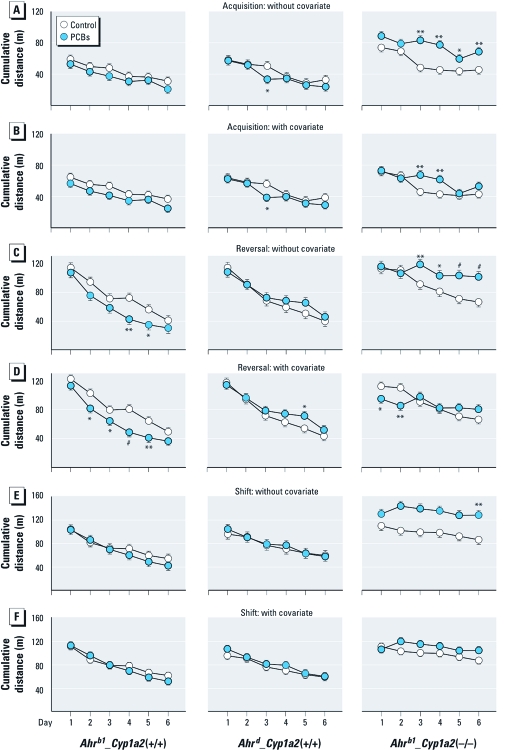
Results of MWM (hidden) test presented as cumulative distance to the platform (mean ± SE). During each phase there were genotype × treatment effects on speed (all *p* < 0.01); therefore, data were analyzed using without (*A,C,E*) and with (*B,D,F*) speed as covariates. (*A *and *B*) Acquisition (southwest platform). (*C *and *D*) Reversal (northeast platform). (*E* and *F*) Shift (northwest platform). See Supplemental Material, Table S2 (http://dx.doi.org/10.1289/ehp.1002965). *p < 0.05, **p < 0.01, and ^#^*p* < 0.001 compared with control.

*MWM, hidden platform.* Latency, path length, and cumulative distance showed the same pattern; therefore, only cumulative distance is shown (Figure 4). During all phases (acquisition, reversal, and shift), PCB-treated mice swam more slowly than did controls regardless of genotype. Therefore, we analyzed the data without and with adjustment for swim speed. Among the *Ahr^b1^_Cyp1a2*(+/+) mice, PCB treatment affected performance during reversal learning (Figure 4C,D). With and without adjustment for speed, the PCB-treated mice showed improved performance (i.e., shorter cumulative distances to reach the goal), compared with controls. Among *Ahr^d^_Cyp1a2*(+/+) mice, PCB treatment was associated with minor differences: on day 3 of acquisition, PCB-treated mice showed shorter cumulative distance than did controls, and on day 5 PCB-treated mice had increased cumulative distance, which is significant with covariate adjustment (Figure 4A–D).

The most striking effects occurred among *Ahr^b1^_Cyp1a2*(–/–) mice (Figure 4): The PCB-treated group showed impaired acquisition, reversal, and shift learning without adjustment for speed (Figure 4A,C,E). During acquisition, adjustment for speed reduced the magnitude and number of days that were significant but did not eliminate the deficit (Figure 4B). During reversal, adjustment for speed eliminated and reversed the deficit on days 1 and 2 (Figure 4D), indicating that swimming ability of the PCB-treated group might account for this effect. Similarly, during the shift, impairment in the PCB-treated group was eliminated after adjustment for swim speed (Figure 4F).

Variations were seen between day and sex when they were included in the analyses along with genotype and treatment. PCB treatment was associated with better performance in both *Cyp1a2*(+/+) lines but with poorer performance in the *Ahr^b1^_Cyp1a2*(–/–) line. Analysis showed that PCB treatment primarily caused differences among females [see Supplemental Material, Figure S1 (http://dx.doi.org/10.1289/ehp.1002965)].

**Figure 5:**
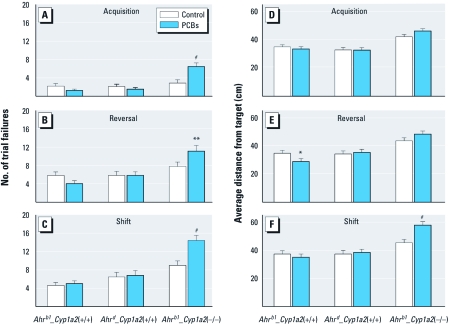
MWM trial failure (trials during which animals reached the time limit; *A*–*C*) and probe trials (average distance from the platform site 24 hr after the last platform trial of each phase; *D*–*F*). (*A* and *D*) Acquisition. (*B* and *E*) Reversal. (*E* and *F*) Shift. Values shown are (mean ± SE). *p < 0.05, **p < 0.01, and ^#^*p* < 0.001 compared with control.

*MWM trial failure.* We also analyzed trial failure, which represents the proportion of trials that the mouse reached the 60-sec time limit and had to be removed. Analyses of these data (Figure 5A–C) demonstrate that only PCB-treated *Ahr^b1^_Cyp1a2*(–/–) mice showed increased rates of failure on all three phases (acquisition, reversal, and shift), compared with controls.

*MWM memory.* All measures of probe-trial performance (a measure of spatial memory because the platform has been removed) showed similar patterns; therefore, only average distance to target is presented in Figure 5D–F. We observed no differences on the acquisition probe (Figure 5D). On reversal probe, PCB-treated *Ahr^b1^_Cyp1a2*(+/+) mice had significantly shorter distances to the platform site than did controls (Figure 5E). On shift probe, PCB-treated *Ahr^b1^_Cyp1a2*(–/–) mice had significantly longer distances to the platform site than did controls, consistent with the trial failure data for this group (Figure 5F).

*Methamphetamine challenge.* We retested locomotor activity after a dose of the positive entantiomer (+) of methamphetamine (an indirect dopaminergic agonist). Before the challenge, we observed no differences among *Ahr^d^_Cyp1a2*(+/+) or *Ahr^b1^_Cyp1a2*(–/–) mice [see Supplemental Material, Figure S2B,C (http://dx.doi.org/10.1289/ehp.1002965)]; however, among *Ahr^b1^_Cyp1a2*(+/+) mice, PCB-treated animals were again significantly more active than controls (see Supplemental Material, Figure S2A). After methamphetamine, mice in all groups showed the typical pattern of hyperactivity. No significant differences as a function of PCB treatment were seen among the *Ahr^d^_Cyp1a2*(+/+) mice (see Supplemental Material, Figure S2B). Among *Ahr^b1^_Cyp1a2*(–/–) mice (see Supplemental Material, Figure S2C), we observed small differences in the PCB-treated group compared with controls. Because of differences in predrug activity in PCB-treated *Ahr^b1^_Cyp1a2*(+/+) mice (see Supplemental Material, Figure S2A, left), the postchallenge analysis used the last 10 min of the prechallenge data as a covariate; ANCOVA indicated only one significant PCB-related difference (during the 80-min test interval; data not shown).

**Figure 6:**
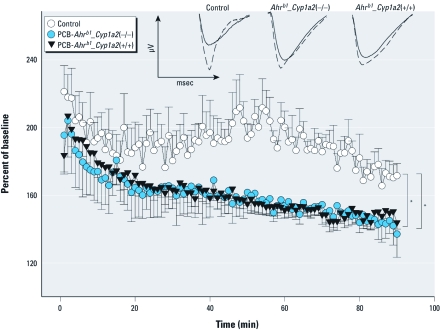
LTP results shown as the percentage of baseline (mean ± SE). The inset shows the EPSP slope in CA1 region slices; the solid line represents the baseline response, and the dashed line represents the response 30 min following tetanus [prolonged membrane response to the electrical stimulation; 100 Hz in 10 bursts (four pulses per burst) delivered at a burst frequency of 5 Hz for 2 sec]. No additional units are possible on the axes because the absolute amplitudes and times vary because of the different specific cells being measured (graphs in the inset represent the wave form, not the exact values of any given curve). No differences between the groups were seen within treatments for response to stimulus or baseline response (*n* = 6, with all mice originating from separate litters). ANOVA showed an effect of group (treatment/genotype) [*F*(2, 13) = 4.87; *p* < 0.05] and no group × time interaction. Dunnett tests comparing PCB-treated groups with controls showed that both the *Ahr^b1^_Cyp1a2*(+/+) and *Ahr^b1^_Cyp1a2*(–/–) PCB-treated groups exhibited significantly decreased LTP induction compared with untreated *Ahr^b1^_Cyp1a2*(+/+) controls. *p < 0.05 compared with control averaged across time.

*LTP.* Another way to examine whether PCB treatment alters neuroplasticity is LTP induction. LTP in the CA1 region of the hippocampus is a cellular correlate of spatial learning and memory (Pavlides et al. 1991). We restricted our analysis to *Ahr^b1^_Cyp1a2*(+/+) vehicle-treated controls and the two PCB-treated groups that showed the greatest difference in MWM tests compared with control wild-type, PCB-treated *Ahr^b1^_Cyp1a2*(–/–) and PCB-treated *Ahr^b1^_Cyp1a2*(+/+) mice. In the presence or absence of CYP1A2, PCB treatment significantly impaired LTP in the CA1 region compared with *Ahr^b1^_Cyp1a2*(+/+) controls (Figure 6), confirming that PCB treatment early in life alters neuroplasticity irrespective of the *Cyp1a2* genotype.

*Monoamine neurotransmitter levels.* We observed no differences in monoamine levels in hippocampus or prefrontal cortex among genotypes, regardless of treatment. In the neostriatum, DA levels were lower in PCB-treated versus control *Ahr^d^_Cyp1a2*(+/+) mice (*p* < 0.01); DA levels were significantly lower in PCB-treated *Ahr^b1^_Cyp1a2*(–/–) than in PCB-treated *Ahr^b1^_Cyp1a2*(+/+) mice (*p* < 0.01). For the latter, DA levels were lower in PCB-treated than in control *Ahr^d^_Cyp1a2*(+/+) mice (*p* < 0.01); DA levels were significantly lower in PCB-treated *Ahr^b1^_Cyp1a2*(–/–) than in PCB-treated *Ahr^b1^_Cyp1a2*(+/+) mice (*p* < 0.01; data available upon request).

We observed no structural abnormalities or differences in size, shape, or appearance of the hippocampus, prefrontal cortex, or neostriatum in hematoxylin-and-eosin–stained sections (data not shown).

*Plasma corticosterone levels.* No PCB-related differences were found in plasma corticosterone levels (data not shown).

## Discussion

Since the earliest report of cognitive dysfunction in Yusho and Yu-Cheng PCB poison victims (Abe et al. 1975), there have been attempts to identify the individual congeners responsible for various toxic end points reported, including neurotoxicity (Ryan et al. 1990). The controversy remains unsettled: Some argue that only noncoplanar PCBs have neurotoxic effects (Rice 1999), whereas others report neurotoxic effects after exposure to coplanar PCBs, dioxins, and related AHR ligands (Amin et al. 2000; Seegal et al. 2005).

Others have used commercial mixtures (Branchi et al. 2005; Chishti et al. 1996) or laboratory-developed mixtures (Hamm et al. 2003; Kostyniak et al. 2005). However, even a mixture having the same name (e.g., Aroclor 1254) and expected chemical composition can vary from batch to batch (Kodavanti et al. 2001). The controversy is best summed up in a review by Ulbrich and Stahlmann (2004), in which the authors documented attempts to model PCB-induced neurotoxicity in rodents. Wide variation in dosing concentrations, routes of administration, and animal models has resulted in variability and results that are not always reproducible.

Data from the present study suggest that both AHR and CYP1A2 play important roles in response to an environmentally relevant mixture of PCB congeners (three coplanar and five noncoplanar). The presence of high-affinity AHR decreases the amount of exposure of offspring to coplanar congeners via inducible P450-mediated detoxication pathways (Nebert et al. 2004), whereas maternal CYP1A2 sequesters coplanar PCBs, thereby diminishing the level of their exposure (Dragin et al. 2006).

Our behavioral phenotyping uncovered significant differences at many levels involving the effects of developmental PCB exposure associated with the *Ahr* and *Cyp1a2* genotypes. Genetic background influences behavior (Crawley et al. 1997; Jacobson and Cryan 2007), but differences in genetic background were decreased as contributing factors in the present study because all lines were backcrossed at least eight generations into B6 mice. Nonetheless, we found significant genotype effects independent of treatment for several tests, including the elevated zero maze, locomotor activity, ASR, and MWM. For example, among controls, *Ahr^b1^_Cyp1a2*(+/+) mice showed fewer zone crossings in the elevated zero maze than did mice of the other genotypes (Figure 1C). In addition, *Ahr^b1^_Cyp1a2*(–/–) control mice exhibited longer latencies in the MWM (Figure 3) and higher overall levels of locomotor activity (Figure 2A) compared with controls of the other genotypes.

Effects of PCB were evident in all tests, reinforcing what had been previously reported with PCB congeners. Interestingly, PCB-treated *Ahr^b1^_Cyp1a2*(+/+) mice showed improved MWM performance in reversal (Figure 4) and reversal probe (Figure 5), suggesting that coplanar-PCB–mediated AHR activation can have beneficial effects on selected aspects of learning. Previous studies have shown that rats exposed to 2,3,7,8-tetrachlorodibenzo-*p*-dioxin (TCDD) or coplanar PCBs make fewer errors in the radial-arm maze (Schantz et al. 1996; Seo et al. 1999; Widholm 2003). The results in the present study support the finding that AHR plays an important role in mammalian CNS development. Indeed, even with no apparent ligand-binding properties, AHR analogs in *Caenorhabditis elegans* (Qin and Powell-Coffman 2004) and *Drosophila* (Crews and Brenman 2006) have been demonstrated to be associated with neuronal development.

The behavioral deficits that we observed in the present study are consistent with the hypothesis that maternal hepatic CYP1A2 protects against PCB-induced developmental neurotoxicity in offspring. In a previous study, Dragin et al. (2006) reported that maternal hepatic CYP1A2 and maternal hepatic human CYP1A2 (in place of the mouse analogous protein) provided protection from TCDD-induced cleft palate and hydronephrosis, and that absence of maternal CYP1A2 increased sensitivity to TCDD-induced birth defects. Another study (Curran et al. 2011) showed that maternal hepatic CYP1A2 protected offspring of mothers that received the same PCB mixture used in the present study from PCB-induced toxicity.

Only PCB-treated *Ahr^b1^_Cyp1a2*(–/–) mice showed impairment in novel object recognition (Figure 1D). These data are consistent with human studies (Jacobson et al. 1985; Kilburn 2000), one of which showed deficits on the Fagan test of novel object recognition (Jacobson et al. 1985). As noted above, all human populations display a > 60-fold gradient ranging from low to high CYP1A2 basal levels; however, no human study has specifically assessed CYP1A2 phenotype as a risk factor for PCB neurotoxicity. During MWM testing, we also uncovered spatial learning and memory deficits associated with PCB exposure. PCB-treated *Ahr^b1^_Cyp1a2*(–/–) mice took longer to learn the cued platform than did untreated controls (Figure 3) but only on days 2 and 3, indicating that effects were not a result of treatment-related visual impairment that might interfere with spatial learning during hidden platform testing. PCB-treated *Ahr^b1^_Cyp1a2*(–/–) mice showed deficits in all three phases of hidden-platform testing (Figures 4 and 5). These deficits were more pronounced as the difficulty of the task increased, but some differences between treated and control mice were reduced when we adjusted the cumulative distance parameter for swim speed. This occurred despite the fact that the cumulative distance parameter is less affected by swim speed than is latency (time needed to find platform), suggesting that covariate analysis (with swim speed as the covariate) may have overadjusted this index, perhaps because swim speed and learning were affected simultaneously, making the separation of the two factors imperfect. Most important, the failure rate in PCB-treated *Ahr^b1^_Cyp1a2*(–/–) mice was significantly higher than in all other groups, arguing against an effect mediated by swim speed alone.

In addition, relative to controls, the baseline ASR was lower in PCB-exposed *Ahr^b1^_Cyp1a2*(–/–) and *Ahr^b1^_Cyp1a2*(+/+) mice but not in *Ahr^d^_Cyp1a2*(+/+) mice, indicating that both AHR and CYP1A2 are critical for proper development of this defensive reflex. In a study of Long-Evans rats, Goldey et al. (1995) reported that developmental Aroclor 1254 exposure through PND21 reduced ASR at PND24 but not during adulthood. In a follow-up experiment, a decreased ASR at PND23 was replicated, but the ASR was increased in the adult offspring (Goldey and Crofton 1998). In a more recent study, developmental PCB153 exposure did not affect ASR in Wistar rats (Gralewicz et al. 2009).

Our finding that PCB alters LTP is consistent with previous findings. We found impaired LTP in the hippocampus (Figure 6), as have others (Altmann et al. 2001; Carpenter et al. 2002; Gilbert and Crofton 1999; Gilbert et al. 2000), which suggests that this region is particularly vulnerable to developmental PCB exposure.

Alterations in DA in the neostriatum found in the present study have also been reported previously. Coplanar PCB congeners appear to increase DA, whereas noncoplanar congeners lead to decreased DA levels (Seegal et al. 1997).

## Conclusion

Developmental exposure to an environmentally relevant mixture of coplanar and noncoplanar PCBs was associated with learning and memory deficits in genetically susceptible *Ahr^b1^_Cyp1a2*(–/–) mice; in mice having normal basal and inducible CYP1A2 expression, these effects were significantly decreased. In addition, developmental exposure to AHR agonists appears to improve spatial learning and memory in *Ahr^b1^_Cyp1a2*(+/+) mice in the presence of maternal CYP1A2.

We have generated a novel mouse model for studying genetic susceptibility to PCB-induced neurotoxicity, which is relevant to at-risk human populations. As noted above, humans display > 12-fold variability in AHR affinity and > 60-fold differences in hepatic CYP1A2 basal levels (Nebert et al. 2004). This means that a highly exposed mother with genetic resistance (i.e., high levels of hepatic CYP1A2, both basal and PCB induced) might have a normal child, whereas a less exposed mother who is genetically susceptible (with low levels of hepatic CYP1A2, both basal and PCB induced) could have a child with developmental delays despite lower PCB exposure. AHR inducibility influences both hepatic levels of CYP1A2 and clearance of lower-molecular-weight planar and non coplanar PCBs. Therefore, ultimately, the risk of PCB-induced neurotoxicity must account for both CYP1A2 and AHR variability.

## Supplemental Material

(240 KB) PDF
