# Supplementary material for: In Utero and Lactational Exposure to PCBs in Mice: Adult Offspring Show Altered Learning and Memory Depending on Cyp1a2 and Ahr Genotypes
Source: Environ Health Perspect. 2011 May 13;119(9):1286–93. doi: 10.1289/ehp.1002965 (PMC3230394; doi:10.1289/ehp.1002965)
Supplement: (240 KB) PDF [file ehp.1002965.s001.pdf]

SUPPLEMENTARY MATERIAL

In Utero and Lactational Exposure to PCBs in Mice: Adult Offspring Show Altered Learning and Memory Depending on *Cyp1a2* and *Ahr* Genotypes

Christine P. Curran[1,*], Daniel W. Nebert[1,**], Mary Beth Genter[1], Krishna V. Patel[1], Tori L. Schafer[2], Matthew R. Skelton[2], Michael T. Williams[2], and Charles V. Vorhees[2]

[1]*Department of Environmental Health, and Center for Environmental Genetics (***CEG***)*
University of Cincinnati Medical Center, Cincinnati, Ohio 45267-0056

[2]*Department of Pediatrics, Division of Neurology, University of Cincinnati Medical Center, and Cincinnati Children's Research Foundation, Cincinnati, Ohio 45229*

*Current address for C. P. Curran**:** *Department of Biological Sciences, Northern Kentucky University, Highland Heights, KY 41099*

****Correspondence**:** Daniel W Nebert, MD, Professor, Kettering Laboratory, P.O. Box 670056, Cincinnati OH 45267-0056. Tele 513-558-2619; Fax 513-558-4397. E-mail dan.nebert@uc.edu .

TABLE OF CONTENTS

Materials and Methods	pages 3–4
Statistical Results	pages 5–7
References	page 8
Table S1	page 9
Table S2	pages 10–11
Figure legends	page 12
Figure S1	page 13
Figure S2	page 14

Materials and Methods

Morris Water Maze (MWM). Testing was conducted over 4 weeks, beginning with cued trials (Week-1) followed by three phases of hidden-platform testing (Weeks-2 to -4). The apparatus consisted of a 122-cm-diameter stainless steel tank filled with water (21 ± 1°C) and nontoxic white tempera paint. The platforms were made of white acrylic covered with a white screen to aid traction. The walls of the tank were painted flat white. Platform and start positions are listed in **Table S2**. On cued trials the location of the submerged platform was made visible using an orange ball mounted on a brass pole inserted in the center of the platform. Black curtains were drawn around the tank to obscure distal cues. Each trial lasted up to 60 s. Mice not finding the platform on a given trial were placed on it for 5 s. The inter-trial interval was 15 s. Six trials were given on Day-1 using a fixed-start-and-platform location. This procedure is designed to teach mice to swim directly to the marker, that the platform is the goal, and to remain on it until removed. Previous experience has shown that in mice this initial training improves platform-training on all subsequent phases of the test. Two trials per day were given on Days-2 to -6 with random start and platform locations. The inter-trial interval was 30 s. One mouse that failed to find the platform on all 6 days of cued testing was excluded from further testing. Therefore, mice received only 10 trials of randomized goal training to teach them to rely only on the visual cue of the marker to find the platform.

For hidden-platform trials, curtains were open and visual cues were present on the walls and throughout the room. The platform was submerged 1 cm below the water. Three different platform sizes were used during acquisition, reversal, and shift of 10, 7 and 5 cm, respectively. For acquisition the platform was placed in the SW quadrant, for reversal in the NE quadrant, and for shift in the NW quadrant. "North" is arbitrarily defined as the point opposite the investigator. During each phase there were 6 days of platform trials (four trials per day). Mice that did not find the platform within 60 s were placed on it for 5 s. The inter-trial interval was 15 s. On day-7 a single 30-s probe trial was given to each animal with no platform present and a novel start location. See (Vorhees and Williams 2006) for further details. One mouse stopped searching and floated for all trials for 5 consecutive days and was excluded from further testing and data analysis.

Long-term potentiation. Electrophysiology was performed using the MED64 multi-electrode array (Alpha Med Sciences; Kadoma, Japan) as described previously (Shimono et al. 2002). Parasagittal sections (350 μm) were taken from the hippocampus of PND30 to PND35 mice and transferred to the recording chamber. Pair-pulse stimuli were delivered at the CA1 region and excitatory post-synaptic potentials (**EPSPs**) were recorded until stable baseline slopes were observed for a minimum of 10 min. Stimuli were determined by determining a maximum response from the slice and using a stimulus that produced 40% of the maximum observed response. The stimulus varied between 40-80 μA, depending on the adhesion of the slice and location of electrode placement. The slope of resulting EPSPs was recorded for 90 min following

a theta burst stimulation (5 Hz for 2 s) at the same intensity of the baseline stimulation. Sections were recorded in duplicate for each animal. Data were averaged per animal for analysis (Alpha Med Sciences).

Monoamine neurotransmitter assay. Mice were killed by decapitation on ~PND100 and brain regions dissected on ice as follows**:** hippocampus, caudate-putamen, prefrontal cortex, and hypothalamus. Tissues were snap-frozen on dry ice, weighed, and stored at –80°C. The analysis included quantification of dopamine (**DA**), metabolite 3,4-dihydroxyphenylacetic acid (**DOPAC**), serotonin (**5-HT**), and 5-hydroxyindoleacetic acid (**5-HIAA**).

Each sample was homogenized in 50 volumes of 0.2 N perchloric acid, using a Dounce glass homogenizer, and centrifuged at 20,000 RCF for 5 min. Aliquots (20 μL) of supernatant were injected onto a Varian Microsorb C18 column (5-μm, 250 x 4.6 mm) using a Waters 717-plus auto-sampler. The column was connected to an LC-4B amperometric detector (Bioanalytical Systems; West Lafayette, IN) with a reference electrode at + 0.59 V oxidation potential. The mobile phase contained 35 mM citric acid, 54 mM sodium acetate, 50 mg/L disodium ethylenediamine tetraacetate (**EDTA**), 80 mg/L 1-octanesulfonic acid sodium salt, and 8.5% acetonitrile, pH 3.2**;** the flow rate was 1 mL per min. Chromatograms were integrated using Empower® (Waters, Inc.; Milford, MA), and neurotransmitter concentrations were calculated from standard curves.

Statistical Results

Elevated zero maze and novel object recognition **(Fig. 1)**. Percent time in open**:** genotype [F(2,161) = 3.16, *P* <0.05] only. Head dips**:** genotype trend [F(2,161) = 2.54, *P* = 0.09] and interaction of genotype × treatment [F(2,161) = 3.47, *P* <0.05]. Slice ANOVAs of the interaction showed no effect for genotype but trends toward reduced head dips in the PCB-treated Ahr^{d}*_Cyp1a2(+/+)* group (*P* = 0.06) and increased head dips in the PCB-treated Ahr^{b1}*_Cyp1a2(+/+)* group (*P* = 0.09) compared with controls. Zone crossings**:** genotype [F(2,161) = 9.41, *P* <0.0001] and genotype × treatment interaction [F(2,161) = 7.44, *P* <0.001]. Slice ANOVAs of interaction showed PCB-treated Ahr^{d}*_Cyp1a2(+/+)* group crossed fewer zones (*P* <0.05) and the PCB-treated Ahr^{b1}*_Cyp1a2(+/+)* group crossed more zones than controls (*P* <0.01). Novel object recognition**:** Data are presented as the mean percentage of time spent exploring the novel object. No genotype or treatment effects were found during familiarization. During memory testing, there was a genotype × treatment [F(2,187) = 4.49, *P* <0.02] interaction. Slice ANOVAs showed that the PCB-treated Ahr^{b1}*_Cyp1a2(–/–)* mice exhibited less preference for the novel object than controls.

Locomotor Activity and ASR/PPI **(Fig. 2).** Locomotor horizontal activity**:** genotype [F(2,201) = 6.40, *P* <0.01], genotype × interval [F(10,835) = 2.14, *P* <0.02], and genotype × treatment × interval [F(10,835) = 2.21, *P* <0.02]. Slice ANOVA of the 3-way interaction showed no genotype × treatment effects among Ahr^{d}*_Cyp1a2(+/+) or* Ahr^{b1}*_Cyp1a2(–/–)* mice. For Ahr^{b1}*_Cyp1a2(+/+)* mice, the PCB-treated group was more active during three of six intervals, compared with controls. Startle amplitude**:** genotype [F(2,201) = 6.68, *P* <0.01], treatment [F(1,201) = 7.29, *P* <0.01], genotype × treatment [F(2,201) = 6.30, *P* <0.01], genotype × prepulse [F(4,402) = 9.27, *P* <0.0001], treatment × prepulse [F(2,402) = 7.09, *P* <0.001], and genotype × treatment × prepulse [F(4,402) = 4.40, *P* <0.01]. Slice ANOVAs of the 3-way interaction showed that effects occurred only on no-prepulse trials. PCB-treated Ahr^{b1}*_Cyp1a2(+/+)* mice had reduced startle compared with controls (*P* <0.0001)**;** the PCB-treated Ahr^{b1}*_Cyp1a2(–/–)* group demonstrated a reduced startle compared with controls (*P* <0.0001)**;** PCB-treated Ahr^{d}*_Cyp1a2(+/+)* mice showed a trend toward increased startle compared with controls (*P* = 0.08). In addition, the genotype main effect was caused by the lower startle amplitude in the Ahr^{b1}*_Cyp1a2(–/–)* group, compared with the other genotypes.

MWM cued **(Fig. 3)**. Day-1 (fixed platform/fixed start)**:** No effects (not shown). (***A***) Days-2 to -6 (variable platform/variable start)**;** latency**:** genotype [F(2,204) = 22.61, *P* <0.0001], genotype × treatment [F(2,204) = 4.69, *P* <0.01], genotype × day [F(8,816) = 3.37, *P* <0.001], treatment × day [F(4,816) = 2.45, *P* <0.05], with trends for treatment [F(1,204) = 3.36, *P* = 0.07] and genotype × treatment × sex × day interaction [F(8,816) = 1.69, *P* = 0.10]. Slice ANOVAs of the genotype × treatment interaction showed effects only among the Ahr^{b1}*_Cyp1a2(–/–)* mice in which the PCB-treated group had longer latencies to

find the platform than controls. (***B***) Further analysis revealed that the PCB-treated group swam more slowly only on Days 2 & 3**;** thereafter (Days 4-6) they performed at control levels.

MWM hidden platform (**Fig. 4**). (***A***) Acquisition**:** ANOVA showed effects of genotype [F(2,206) = 26.09, *P* <0.0001], genotype × treatment [F(2,206) = 7.74, *P* <0.001], genotype × treatment × day [F(10,854) = 2.13, *P* <0.05], genotype × treatment × day × sex [F(10,854) = 2.24, *P* <0.02], and a genotype × treatment × sex trend [F(2,206) = 2.85, *P* <0.07)]. (***B***) Acquisition**:** ANCOVA showed a similar pattern with effects of genotype [F(2,206) = 6.51, *P* <0.002], genotype × treatment [F(2,206) = 3.52, *P* <0.05], genotype × treatment × day [F(10,858) = 2.13, *P* <0.05], and genotype × treatment × day × sex [F(10,858) = 2.24, *P* <0.05]. Slice ANOVAs showed in both analyses that interactions with day occurred in Ahr^{b1}*_Cyp1a2(–/–)* and Ahr^{d}*_Cyp1a2(+/+)* mice. In the Ahr^{b1}*_Cyp1a2(–/–)* mice the effects occurred on Days 3-6 without the covariate and on Days 3-4 with the covariate**;** in both cases the PCB-treated group swam longer distances to the platform than vehicle-treated controls. In Ahr^{d}*_Cyp1a2(+/+)* mice, the only effect was on Day 3. With or without the covariate, on Day 3 the PCB-treated group exhibited reduced distance to the platform compared to vehicle-treated controls. (***C***) Reversal**:** ANOVA showed effects of genotype [F(2,206) = 19.45, *P* <0.0001], genotype × treatment [F(2,206) = 5.25, *P* <0.01], genotype × day [F(10,862) = 5.45, *P* <0.0001], treatment × day [F(5,783) = 3.10, *P* <0.01], genotype × treatment × day [F(10,862) = 2.02, *P* <0.05], and treatment × sex × day [F(5,783) = 2.37, *P* <0.05]. (***D***) Reversal**:** ANCOVA showed the same factors to be significant, except that the covariate decreased the F-values for main effects. The significant factors were genotype, F = 6.24, *P* <0.01, genotype × treatment, F = 4.81, *P* <0.01, genotype × day, F = 5.46, *P* <0.0001, treatment × day, F = 3.10, *P* <0.01, genotype × treatment × day, F = 2.02, *P* <0.05, and genotype × treatment × sex × day, F = 2.37, *P* <0.05 (degrees of freedom same as above). Slice-effect ANOVAs showed effects in Ahr^{b1}*_Cyp1a2(+/+)* and Ahr^{b1}*_Cyp1a2(–/–)* mice without the covariate and in all three genotypes with the covariate. Without the covariate, among the Ahr^{b1}*_Cyp1a2(+/+)* mice, the PCB-treated group showed decreased distance to the platform on Days 4 & 5**;** with the covariate, the PCB-treated group showed shorter distances on Days 2, 4 & 5. Without the covariate, the Ahr^{d}*_Cyp1a2(+/+)* mice showed no significant effects**;** with the covariate, the PCB-treated group swam longer distances to the platform on Day 5 compared with controls. For Ahr^{b1}*_Cyp1a2(–/–)* mice, without the covariate the PCB-treated group swam longer distances to the platform on Days 3-6 compared with vehicle-treated controls**;** with the covariate this effect was reversed and PCB-treated had shorter distances to the platform on Days 1 & 2 and no difference on the other days. (***E***) Shift**:** ANOVA showed effects of genotype [F(2,203) = 28.24, *P* <0.0001], treatment [F(1,203) = 3.93, *P* <0.05], genotype × treatment [F(2,203) = 5.41, *P* <0.01], and genotype × day [F(10,858) = 4.82, *P* <0.0001]. (***F***) Shift**:** ANCOVA reduced this to effects of genotype [F(2,204) = 20.03, *P* <0.0001], and genotype × day

[F(10,680) = 4.81, P <0.0001]. Slice-effect ANOVAs on the genotype × treatment interaction showed effects only in *Ahr*^{b1}*_Cyp1a2(–/–)* mice (P <0.001)**;** the PCB-treated group had greater distances to the platform than control.

MWM trial failures and probe trials (**Fig. 5**). For acquisition (***A***), ANOVA showed an effect of genotype [F(2,209) = 17.57, P <0.0001] and genotype × treatment [F(2,209) = 9.84, P <0.001]. For reversal (***B***), there was an effect of genotype [F(2,209) = 14.71, P <0.0001], and genotype × treatment [F(2,209) = 4.35, P <0.02]. For shift (***C***), ANOVA showed three effects**:** genotype [F(2,209) = 32.72, P <0.0001], treatment [F(1,209) = 7.74, P <0.01], and genotype × treatment [F(2,209) = 5.26, P <0.01]. Slice-effect ANOVAs on each phase for each genotype showed effects among only the *Ahr*^{b1}*_Cyp1a2(–/–)* mice**:** acquisition (P <0.0001), reversal (P <0.01), and shift (P <0.0001). In each case, the PCB-treated group had more trial failures than control. (***D***) acquisition-probe**:** ANOVA showed effects of genotype [F(2,203) = 26.74, P <0.0001], and a treatment × sex trend [F(1,203) = 3.10, P =0.08]. (***E***) reversal-probe**:** there were effects of genotype [F(2,203) = 24.35, P <0.0001], and genotype × treatment [F(2,203) = 3.32, P <0.05]. Slice ANOVA on each genotype showed an effect only in *Ahr*^{b1}*_Cyp1a2(+/+)* mice (P <0.05)**;** PCB-treated mice had reduced distance to the platform site, compared with control. (***F***) shift-probe**:** there were effects of genotype [F(2,202) = 25.47, P <0.0001], treatment [F(1,202) = 3.74, P = 0.05], and genotype × treatment [F(2,202) = 5.57, P <0.01]. Slice-effect ANOVAs on the interaction showed effects only in the *Ahr*^{b1}*_Cyp1a2(–/–)* mice (P <0.001) in which PCB-treated mice showed longer distances to the platform site than control. Other measures of probe performance (crossovers, percent time and percent distance in the target quadrant) were similar to average distance findings.

Long-term potentiation **(Fig. 6)**. LTP in brain slices**:** ANOVA showed an effect of group (treatment/genotype) [F(2, 13) = 4.87, P <0.05] and no group × time interaction. Dunnett tests comparing PCB-treated groups with control showed that both the *Ahr*^{b1}*_Cyp1a2(+/+)* and *Ahr*^{b1}*_Cyp1a2(–/–)* PCB-treated groups showed significantly decreased LTP induction compared with controls.

References

Shimono, K., Baudry, M., Ho, L., Taketani, M. and Lynch, G. (2002). Long-term recording of LTP in cultured hippocampal slices. Neural Plast., 9, 249-254.

van den Berg, M., Birnbaum, L. S., Denison, M., De, V. M., Farland, W., Feeley, M., Fiedler, H., Hakansson, H., Hanberg, A., Haws, L., Rose, M., Safe, S., Schrenk, D., Tohyama, C., Tritscher, A., Tuomisto, J., Tysklind, M., Walker, N., and Peterson, R. E. (2006). The 2005 World Health Organization reevaluation of human and Mammalian toxic equivalency factors for dioxins and dioxin-like compounds. *Toxicol. Sci.* 93, 223–241.

Vorhees, C. V., and Williams, M. T. (2006). Morris water maze: procedures for assessing spatial and related forms of learning and memory. *Nat. Protoc.* 1, 848–858

TABLE S1. List of the eight PCB congeners in the mixture used in this study.

PCB congener	Planarity	IUPAC #	Dose / kg	Toxic equivalency factor (TEF)*
**3,3'4,4'-Tetrachlorobiphenyl**	Coplanar	77	5 mg	0.0005
**3,3',4,4',5-Pentachlorobiphenyl**	Coplanar	126	25 μg	0.1
**3,3',4,4',5,5'-Hexachlorobiphenyl**	Coplanar	169	250 μg	0.03
**2,3,3',4,4'-Pentachlorobiphenyl**	Non-coplanar	105	10 mg	0.00003
**2,3',4,4',5-Pentachlorobiphenyl**	Non-coplanar	118	10 mg	0.00003
**2,2',3,4,4',5'-Hexachlorobiphenyl**	Non-coplanar	138	10 mg	0.0005
**2,2',4,4',5,5'-Hexachlorobiphenyl**	Non-coplanar	153	10 mg	0.0005
**2,2',3,4,4',5,5'-Heptachlorobiphenyl**	Non-coplanar	180	10 mg	0.00001

***Listed in (van den Berg *et al.* 2006).**

Table S2. **MWM cued platform positions: North (N) was arbitrarily designated as the location directly opposite the experimenter. First direction listed is the start location and the second is the location of the platform. W = west; E = east; S = south.**

(A) Cued platform (10-cm platform)

	**Trial 1**	**Trial 2**	**Trial 3**	**Trial 4**	**Trial 5**	**Trial 6**
Day 1	W –E	W –E	W –E	W –E	W –E	W –E
Day 2	NW – NE	S – NW				
Day 3	E - SW	S W- NW				
Day 4	NE - SE	N – SW				
Day 5	W - SE	SE – NE				
Day 6	NW - NE	S – NW				

(B) Acquisition (hidden platform). Platform was in the SW quadrant.

	**Trial 1**	**Trial 2**	**Trial 3**	**Trial 4**
Day 1	N	E	SE	NW
Day 2	SE	N	NW	E
Day 3	NW	SE	E	N
Day 4	E	NW	N	SE
Day 5	N	SE	E	NW
Day 6	SE	NW	N	E
***Day 7* (Probe)**	NE			

(C) Reversal (7-cm platform). Platform was in the NE quadrant.

	**Trial 1**	**Trial 2**	**Trial 3**	**Trial 4**
Day 1	S	W	NW	SE
Day 2	NW	S	SE	W
Day 2	SE	NW	W	S
Day 4	W	SE	S	NW
Day 5	S	NW	W	SE
Day 6	NW	SE	S	W
***Day 7* (Probe)**	SW			

Table S2, concluded

(D) Shift (5-cm platform). Platform was in the NW quadrant.

	Trial 1	Trial 2	Trial 3	Trial 4
Day 1	S	E	NE	SW
Day 2	NE	S	SW	E
Day 3	SW	NE	E	S
Day 4	E	SW	S	NE
Day 5	S	NE	E	SW
Day 6	NE	E	SW	S
***Day 7* (Probe)**	SE			

Figure Legends

Figure S1. **MWM (acquisition by sex). Based on the significant genotype × treatment × sex × day interactions noted in Figure 6, slice-effect ANOVAs were performed for each day separately on males and females. In males, effects were seen only in the *Ahr^{b1}_Cyp1a2(–/–)* mice on Days 3& 6 in which PCB-treated swam longer distances to the platform. For females, all three genotypes showed significant effects. Among *Ahr^{b1}_Cyp1a2(+/+)* mice, effects were seen on Days 2 & 3 in which the PCB-treated group had shorter distances to the platform; this was also seen in *Ahr^{d}_Cyp1a2(+/+)* mice on Day 3 with the PCB-treated group showing shorter distance to the platform. In *Ahr^{b1}_Cyp1a2(–/–)* mice, the female PCB-treated group had longer distances to the platform than control on Days 1-4 & 6. ***P* <0.05, #*P* <0.01 vs. control.**

Figure S2. Locomotor activity with drug challenge. Horizontal beam breaks prior to and after *(+)*-methamphetamine (1 mg/kg) challenge. **P* <0.05 vs control. ANOVA showed a treatment effect in *Ahr^{b1}_Cyp1a2(+/+)* mice [F(1,58.6) = 16.41; *P* <0.001] but not in *Ahr^{d}_Cyp1a2(+/+)* or *Ahr^{b1}_Cyp1a2(–/–)* mice. In order to account for these differences in post-challenge performance, we analyzed data by ANCOVA using as the covariate the last 10 min of the pre-challenge performance; this analysis showed effects of genotype × interval (F(22,1870) = 4.95; *P* <0.001] and treatment × interval [F(11,1870) = 1.78; *P*=0.05]. Slice ANOVAs on genotype for each interval showed that in *Ahr^{b1}_Cyp1a2(+/+)* mice, the PCB-treated group was more active at the 80-min interval (*P* = 0.08); in *Ahr^{b1}_Cyp1a2(–/–)* mice the PCB-treated group under-responded to the drug at 30 min (*P* = 0.08) and over-responded at 120 min (*P* <0.05).

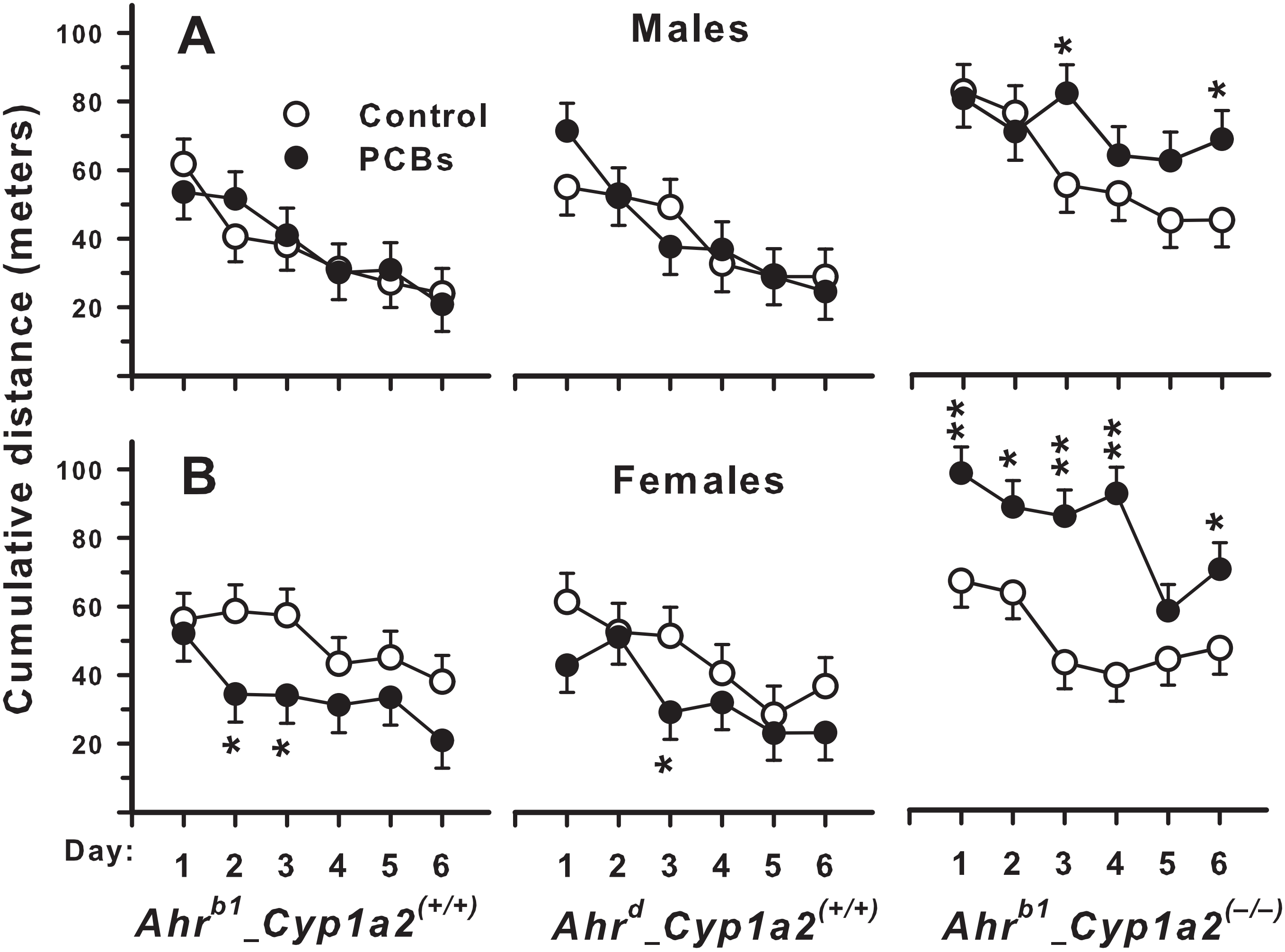

A
Males
Control
PCBs
B
Females
Cumulative distance (meters)
100
80
60
40
20
Day:
1 2 3 4 5 6
*
Ahr^b1_Cyp1a2^(+/+)
Ahr^d_Cyp1a2^(+/+)
Ahr^b1_Cyp1a2^(−/−)

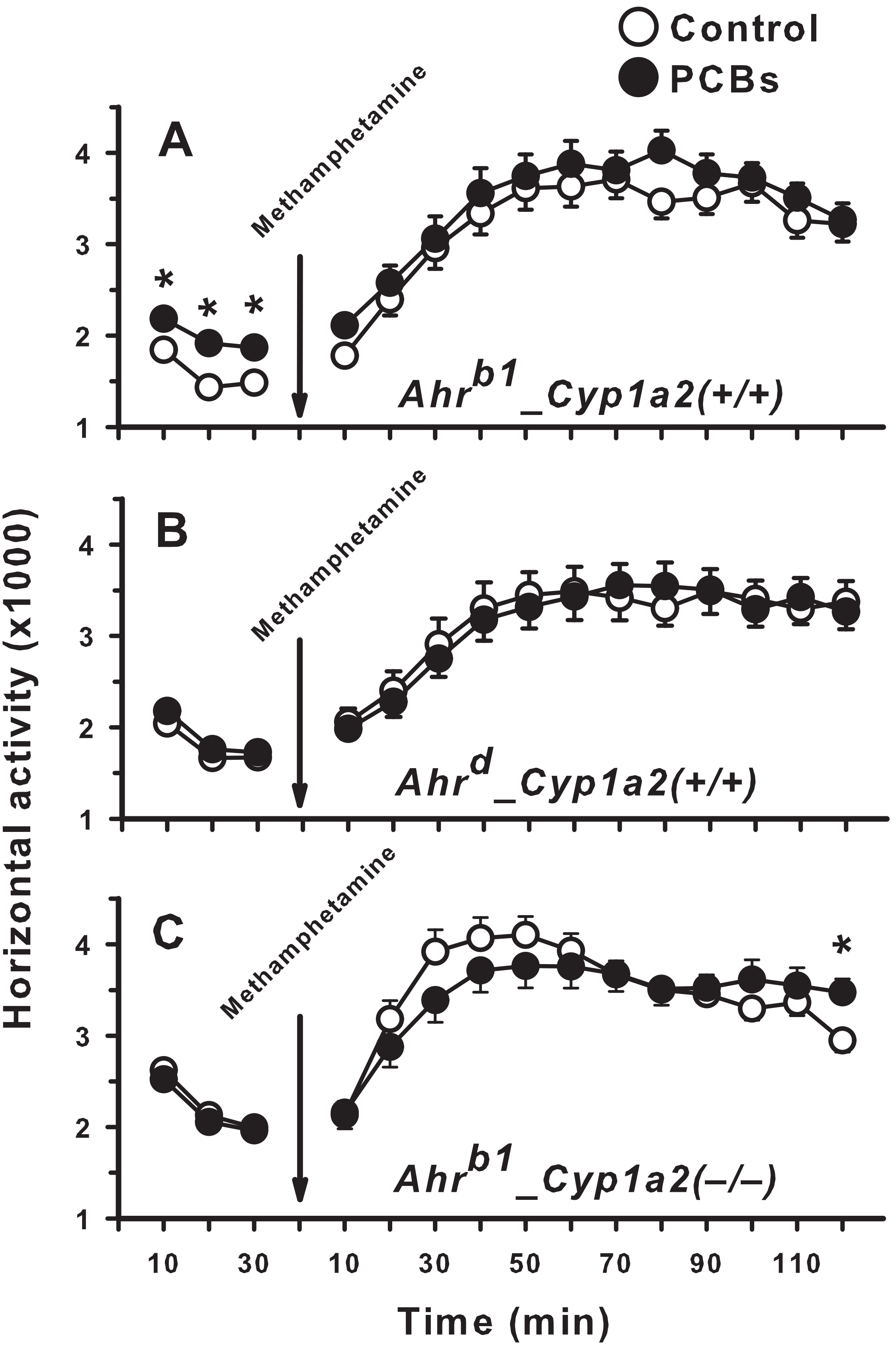
Control
PCBs
A
Methamphetamine
*
*
*
Ahr^b1_Cyp1a2(+/+)
B
Methamphetamine
Ahr^d_Cyp1a2(+/+)
C
Methamphetamine
*
Ahr^b1_Cyp1a2(–/–)
Horizontal activity (x1000)
1
2
3
4
10
30
10
30
50
70
90
110
Time (min)
